# HIV-1 and methamphetamine alter galectins -1, -3, and -9 in human monocyte-derived macrophages

**DOI:** 10.1007/s13365-021-01025-4

**Published:** 2022-02-17

**Authors:** Kinga Grabowska, Katarzyna Macur, Sarah Zieschang, Lubaba Zaman, Nicole Haverland, Andrew Schissel, Brenda Morsey, Howard S. Fox, Pawel Ciborowski

**Affiliations:** 1 Department of Pharmacology and Experimental Neuroscience, School of Medicine, University of Nebraska Medical Center, Omaha, NE USA; 2 Laboratory of Virus Molecular Biology, Intercollegiate Faculty of Biotechnology, University of Gdańsk and Medical University of Gdańsk, Gdańsk, Poland; 3 Core Facility Laboratories, Intercollegiate Faculty of Biotechnology, University of Gdańsk and Medical University of Gdańsk, Gdańsk, Poland

**Keywords:** Galectins, HIV, Innate immunity, Macrophages, Multiple reaction monitoring, Quantitative proteomics

## Abstract

Macrophages are key elements of the innate immune system. Their HIV-1 infection is a complex process that involves multiple interacting factors and various steps and is further altered by exposure of infected cells to methamphetamine (Meth), a common drug of abuse in people living with HIV. This is reflected by dynamic changes in the intracellular and secreted proteomes of these cells. Quantification of these changes poses a challenge for experimental design and associated analytics. In this study, we measured the effect of Meth on expression of intracellular and secreted galectins-1, -3, and -9 in HIV-1 infected human monocyte-derived macrophages (hMDM) using SWATH-MS, which was further followed by MRM targeted mass spectrometry validation. Cells were exposed to Meth either prior to or after infection. Our results are the first to perform comprehensive quantifications of galectins in primary hMDM cells during HIV-1 infection and Meth exposure a building foundation for future studies on the molecular mechanisms underlying cellular pathology of hMDM resulting from viral infection and a drug of abuse—Meth.

**Supplementary information:**

The online version contains supplementary material available at 10.1007/s13365-021-01025-4.

## Introduction

HIV-1 infection has devastating effects at various levels on the function of the entire organism (Niu et al. 2020; Passaro et al. 2015; Saito et al. 2008). The macrophage (mononuclear phagocytes, MP), a key component of the innate immune system, is one of the prime targets of HIV-1 and a reservoir of productive viral infection constituting a vehicle to spread infection to organs. Thus, its impact on the course of disease is central. The complexity of HIV infection and treatment is further exacerbated by using drugs of abuse because two entities (virus and drug of abuse) are involved, the virus and use of drugs of abuse. Treatments are quite different in nature (Liang et al. 2008). Meth also has untoward effects on various physiological systems including the immune system (Papageorgiou et al. 2019) and is one of the frequently used drugs in people living with HIV (PLWH) (Lyons et al. 2013; Mitchell et al. 2006; Pantalone et al. 2010).

MPs survey their milieus and react to contain their pathological environment, including infections, or other sources of inflammation. However, exposure to toxic substances, including Meth, impairs their protective capacities. It has been shown that HIV-1 infection results in changes in MP protein expression (Talloczy et al. 2008); and therefore, the addition of Meth may further impact the functioning of the innate immune system (Kraft-Terry et al. 2009).

Galectins are defined as lectins binding β-galactoside. There have been 15 galectins discovered in mammals, out of which nine, galectin-1, -2, -3, -4, -7, -8, -9, -10, and -12, have been found in humans (https://www.genenames.org/data/genegroup/#!/group/629). Because carbohydrate–protein interactions are generally weak (Laederach and Reilly 2005), dissociation constants for most lectin–monosaccharide interactions are in the millimolar range (Schwarz et al. 1993). It has been shown that the galectin-1 dimer is the most thermodynamically stable of all galectins with dimer dissociation constant of K_d_ ~ 2–7 × 10^−6^ M. Based on biochemical characterization of lectins, galectins, as well as other lectins, enable the formation of transient states, which might support interactions with protein as their ligands (Schwarz et al. 1993).

Although galectins have been postulated as central regulators of the immune system (Brinchmann et al. 2018; Paclik et al. 2011), the mechanism of how they regulate monocyte/macrophage physiology, which constitute the connection between innate and adaptive branches of the immune system, is still unknown. Paclik et al. postulate that galectins uniquely modulate central monocyte/macrophage function (Paclik et al. 2011). The authors showed that galectins link the innate and adaptive immune systems by inhibiting T-cell function via macrophage priming.

**Fig. 1:**
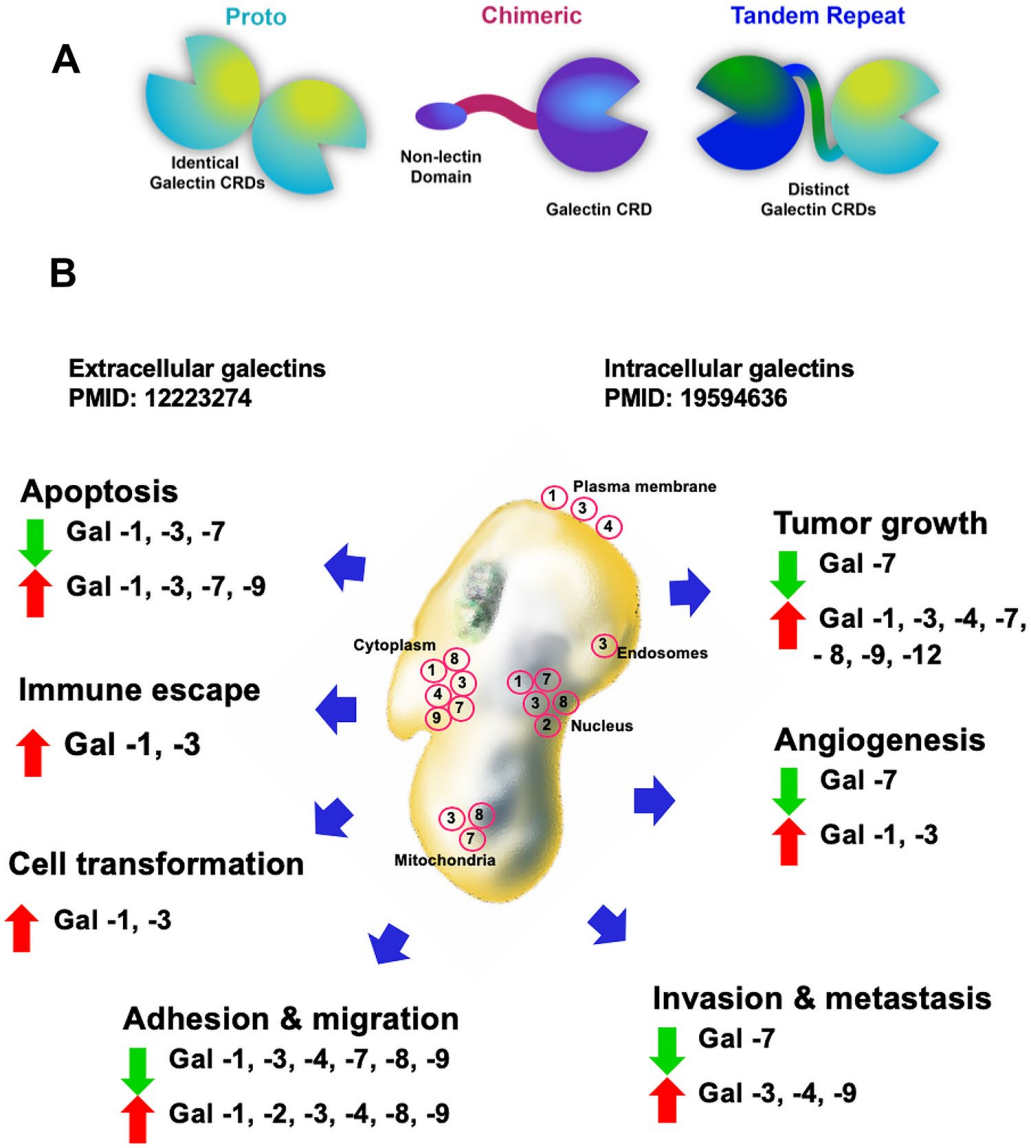
Galectins and their functions in macrophages. (***a***) Types of galectins. (***b***) Summary of functions of galectins in macrophage (adapted from (Johannes et al. 2018; Vladoiu et al. 2014))

As depicted in Fig. 1a, galectins are divided to three subgroups: prototype, chimera type, and tandem repeat type. Galectin-1, galectin-3, and galectin-9 represent prototype, chimeric, and tandem repeat groups, respectively. Regardless of their structure, galectins participate in regulation of many cellular functions in macrophages as presented in Fig. 1b. These are, but not limited to, adhesion and migration, apoptosis, cell transformation, tumor growth, angiogenesis, and immune escape. A wide variety of biological processes have been reported to be related to galectins, such as development, differentiation, morphogenesis, tumor metastasis, apoptosis, and RNA splicing. Nevertheless, the mechanism by which galectins use carbohydrate recognition for their functions is not fully understood.

As galectins themselves and their involvement in HIV-1 infection have been studied to some extent (Shahbaz et al. 2020), the effect of Meth on their expression is represented by very few publications (Parikh et al. 2015; Reynolds et al. 2007). In this study, we present results of the effect of Meth on intracellular and extracellular expression of galectins-1, -3, and -9 in HIV-1 infected human monocyte-derived macrophages (hMDM). Each galectin represents different sub-type of this molecule. Interestingly, we demonstrate that high sensitivity and accuracy methods such as mass spectrometry-based multiple reaction monitoring (MRM) show significant differences in expression, which are not readily detected by screening methods such as sequential window acquisition of all theoretical mass spectra (SWATH-MS).

Galectins are soluble proteins found primarily in the cytosol, nucleus, extracellular matrix, or in circulation. Our immunocytochemical staining shows that an intracellular pool of these three galectins is divided between lysosomes and cytosol. This suggests that each pool of galectin plays different role(s) in cells’ metabolism.

## Materials and methods

### Patients and samples

As reported before (Haverland et al. 2014; Macur et al. 2021), monocytes from healthy human donors were obtained from HIV-1, HIV-2, and hepatitis seronegative donors and differentiated to hMDM. The University of Nebraska Medical Center (UNMC) Institutional Review Board has determined utilizing core facilities from leukapheresis of normal donors does not constitute human subject research as defined in 45CFR46.102 of US Federal Policy for the Protection of Human Subjects. Therefore, this leukapheresis procedure is not subject to federal regulation of human subject research and has been classified as exempt.

### Cell cultures and infections

**Fig. 2:**
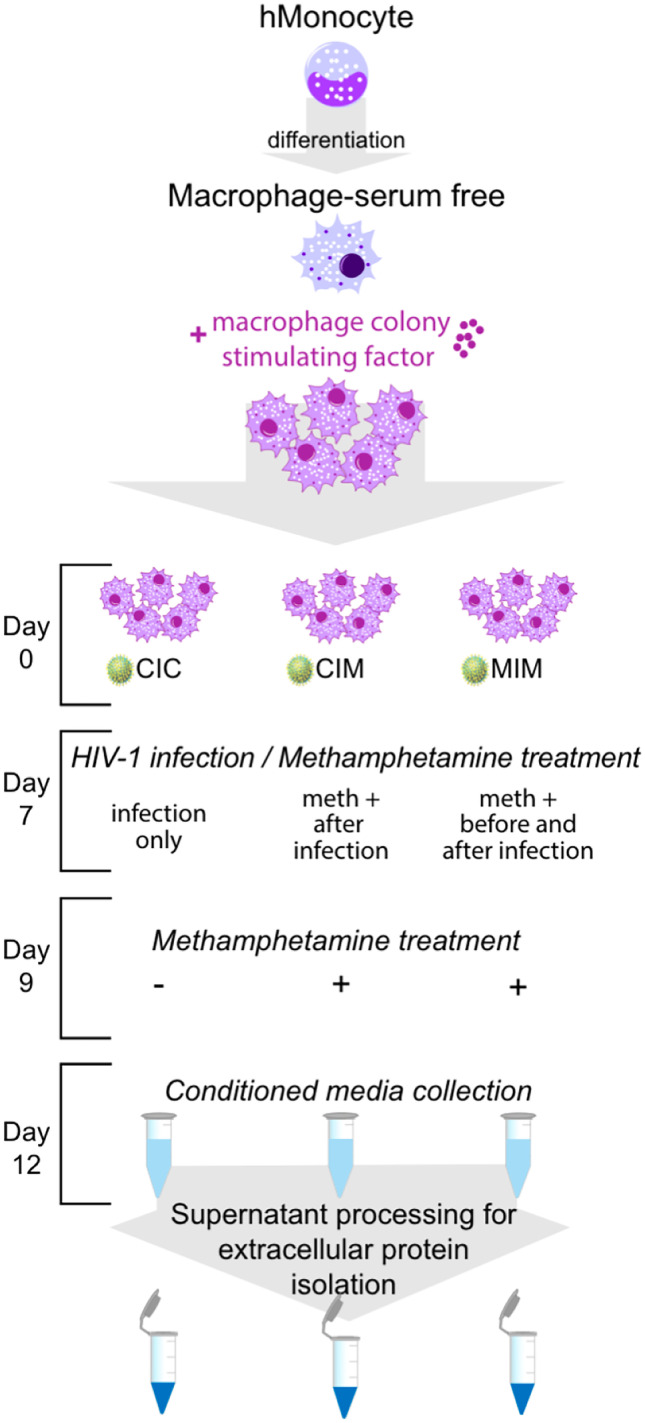
Experimental design. CIC signifies condition in which infection only occurs; CIM represents Meth treatment post-infection. MIM signifies Meth treatment before and after infection. An addition of Meth prior to and after infection (MIM) and after infection (CIM) indicates longer and shorter time of exposure to Meth, respectively

Samples were prepared as described in Macur et al. (2021). Leukapharesed monocytes purified by counter-current centrifugal elutriation were resuspended in the serum-free Macrophage-SFM media (Life-Tech, Inc., Houston, TX, USA) supplemented with 10-mM HEPES (Invitrogen, Carlsbad, CA, USA), 50 mg/mL gentamicin (Invitrogen), 2 µg/mL ciporflaxin (Invitrogen), 1% Nutridoma-SP (Sigma, St. Louis, MO, USA), and 10 ng/mL of recombinant human macrophage colony stimulating factor (MCSF; PeproTech, Rocky Hill, NJ, USA) and then seeded on 6 well culture plates. Cells were cultured for 7 days with half- and complete-media exchange on 3rd and 5th day, respectively, as well as with or without Meth (Sigma) treatment (Meth final concentration 100 µM). On 7th day post-elutriation, media were discarded, and differentiated macrophages were exposed for 4 h to 1 ml of serum-free media containing HIV-1_ADA_ (TCID_50_ = 10^4.36 per ml, as measured on human monocyte-derived macrophages). After 4-h exposure, all media containing HIV-1_ADA_ was removed, cells were washed with serum-free media four times, covered with the fresh serum-free Macrophage-SFM media supplemented with 10-mM HEPES, 50-mg/mL gentamicin, 2-µg/mL ciporflaxin, and 1% Nutridoma-SP (without addition of MSCF) with or without Meth treatment and cultured for a total of 5 days post HIV-1_ADA_. On 3rd day post-infection, a full media exchange (with or without Meth treatment) was performed and the supernatant reserved for a p24 assay (Macur et al. 2021). On 5th day post-infection, conditioned media was collected, spun at 1500 rpm to remove any floating cells and major debris and transferred to the 50-ml tubes. Cells were washed with ice-cold Dulbecco’s phosphate-buffered saline (DPBS; Corning, Manassas, VA, USA); and DPBS with the Halt™ Protease and Phosphatase Inhibitor Cocktail, EDTA-free (Thermo Fisher Scientific, Waltham, MA, USA), was added to each well. Each well was scraped; the cellular suspension was transferred to 15-mL tubes, washed in DPBS with the protease inhibitors, and pelleted by centrifugation. Cell pellets and collected conditioned media of HIV-1 uninfected/Meth untreated (CCC), infected with HIV-1 (CIC), exposed to Meth post-infection (CIM), and treated with Meth before and after HIV-1 infection (MIM) were stored at − 80 °C for further analysis. For further details see Fig. 2.

### RNA isolation and RNAseq

For RNA isolation, the culture medium was removed; cells were washed with ice-cold DPBS; and attached hMDMs were dissolved in Trizol (Invitrogen) and stored at − 80 °C. Trizol MDM extracts were subsequently thawed; chloroform was added to each tube, mixed, incubated at room temperature for 10 min, and further separated by centrifugation. The aqueous phase was transferred to a new tube, and RNA was precipitated by adding isopropyl alcohol then incubated for 20 min at − 20 °C. RNA was then recovered by centrifugation at 12,000 g at 4 °C for 10 min, and the pellet dissolved in RNAse-free water. The RNAeasy clean-up kit (Qiagen, Germantown, MD, USA) was used for cleaning the RNA preparation. Quantity and purity were quantified by absorbance measurement at 260 nm and 230/280 nm absorbance, respectively. Intactness of prepared samples was assessed on a Fragment Analyzer (Agilent Technologies, Ankeny, IA, USA).

The RNA samples then were used for cDNA library construction using TrueSeq mRNA sample prep V2 (Illumina, San Diego, CA, USA) followed by next-generation sequencing performed on the Illumina NextSeq550. The sequencing data was processed and mapped to the human reference genome as described (Macur et al. 2021). For expression calculation and ANOVA analysis, values of Transcripts Per Kilobase Million (TPM) were calculated; and data were imported into the Partek Flow genomic analysis software package (Partek Inc., St. Louis, MO, USA), followed by batch correction for donors using a generalized linear model and statistical analysis by ANOVA.

### Cell lysates and extracellular proteins from conditioned media

Cell lysis was performed as described in our other papers (Burns and Ciborowski 2016; Macur et al. 2021). Samples were digested using the Filter Aided Sample Preparation (FASP) protocol (Wisniewski et al. 2009). Obtained peptides were cleaned by applying Oasis mixed cation exchange procedure according to the manufacturer’s protocol (Waters, Milford, MA, USA). Peptides concentration was measured using a NanoDrop 2000 (Thermo Scientific, Wilmington, DE, USA). Collected supernatants were thawed, supplemented with protease/phosphatase inhibitor cocktail, Triton X-100 (Fisher Scientific, Pittsburg, PA, USA) at a final concentration of 0.1% and after incubation centrifuged at 1500 rpm for 10 min. New supernatants were transferred to the 50 mL tubes and concentrated using Amicon®Ultra-15 centrifugal filter units (Millipore, Bedford, MA, USA) with a membrane cutoff of 3000 MWCO (molecular weight cut-off). Concentrated supernatants were processed for ethanol precipitation procedure, followed by albumin/IgG depletion using Pierce™ Albumin/IgG Removal Kit (Thermo Fisher Scientific). For total protein concentrations measurement Pierce 660 nm Protein Assay (Thermo Fisher Scientific) was applied.

### Liquid chromatography-tandem mass spectrometry analysis

#### NanoLC-SWATH-MS analyses

A nanoLC-MS/MS (nano liquid chromatography–tandem mass spectrometry) technique in a data-independent acquisition (DIA) SWATH (Sequential Windowed Acquisition of All Theoretical Fragment Ion Mass Spectra) mode was applied for full unbiased proteomic profiling of CIC, CIM, and MIM samples from four donors, as described in Macur et al. (2021). The nanoLC-MS/MS system consisted of Eksigent 415 nanoLC (Eksigent, Redwood, CA, USA) coupled with TripleTOF 6600 (SCIEX, Framingham, MA, USA) mass spectrometer with ESI OptiFlow TurboV Ion Source and SteadySpray NanoProbe (SCIEX). The system was controlled by Analyst TF 1.7 software (SCIEX). The CIC, CIM, and MIM samples consisted of aliquots of trypsin-digested hMDM whole cell extracts from all the studied donors for each of the conditions. They were spiked with iRT Kit peptides (Biognosys AG, Zurich, Switzerland) and analyzed by SWATH-MS in five technical replicates. Peptide mixtures (2 µg/injection) were loaded onto the RP-1 Trap Column (General RP, 10 × 0.075 mm; Phenomenex, Torrance, CA, USA) for 10 min (2 µL/min flow rate) and transferred to the bioZen Peptide Polar-C18 separation column (150 × 0.075 mm, 3 μm; Phenomenex) through SecurityLINK Sgl (25 µm × 75 cm, Phenomenex). The chromatographic separation was carried out using the mobile phases A (0.1% formic acid in water) and B (0.1% formic acid in acetonitrile (ACN) (all solvents LC–MS grade) at the flow rate of 300 nL/min. The gradient program started from 3 to 8% B (0–2 min), then linearly increased to 30% B (2–90 min), 40% B (90–100 min) and 80% B (100–105 min); then it was maintained at 80% B (105–110 min) and followed by column re-equilibration at 3% B (112–135 min). The chromatographically separated samples were then ionized by ESI at 3 kV ion spray voltage and analyzed by SWATH-MS in a positive ion mode for 130 min, where the TOF–MS MS1 scan in the m/z range of 400–1000 Da was divided into 100 overlapping variable precursor isolation windows and followed by MS2 scans acquired in a looped product ion mode in 100–1500 m/z range for + 2 to + 5-charged precursors. A pooled CIC-CIM-MIM sample spiked with iRT Kit peptides was also analyzed in four technical replicates using data-dependent acquisition (DDA) in positive ion mode on the same nanoLC-MS/MS system, where up to 30 of the most intense precursor ions were selected from TOF–MS MS1 scan (400–1250 Da) for fragmentation in the 100–1500 m/z range. The nanoLC and ion source set-up in and parameters in DDA analyses were identical to the SWATH-MS analyses. The mass spectrometry DDA and SWATH data from the presented study were deposited to the ProteomeXchange Consortium (Perez-Riverol et al, 2019) (http://proteomecentral.proteomexchange.org) and are available under the PXD023291 dataset identifier. The obtained DDA MS/MS spectra of the pooled CIC-CIM-MIM sample were searched together using ProteinPilot 5.0.1 software (SCIEX) with Paragon Algorithm against a human and HIV reviewed database from UniProt (https://www.uniprot.org/) (downloaded on 09.04.2020) to create a spectral ion library. This spectral ion library (only peptides with confidence threshold of 99% and 1% FDR rate, shared peptides excluded) was then applied for the SWATH data extraction with the use of SWATH 2.0 MicroApp in PeakView 2.2 software (SCIEX). The extracted peak areas for the proteins were then exported to MarkerView 1.2.1 software (SCIEX), normalized, and statistically analyzed using t-Test to compare CIC vs. CIM, CIC vs MIM, and CIM vs MIM conditions. The details of the nanoLC-MS/MS DIA SWATH and DDA MS/MS methods’ settings as well as parameters of the database search, SWTAH data extraction, and statistical analysis with the resulting *p* values (statistical significance at *p* < 0.05) and Log_10_(Fold Change) of proteins quantified in hMDM in CIC vs. CIM, CIM vs. MIM, and CIM vs. MIM conditions, can be found in Macur et al. (2021). Sequence coverage resulting from nanoLC MS/MS analysis for galectins-1, -3, and -9 are shown in Supplemental Fig. S1.

#### LC-MRM analyses

A reverse phase ultra-high-performance LC–MS/MS technique in MRM mode was applied for quantification of galectins (1, 3, 9) in five human donors’ samples. The analyses were performed on the Nexera X2 UHPLC with a SIL-30AC Autosampler (Shimadzu, Kyoto, Japan) coupled with the QTRAP 6500 mass spectrometer (SCIEX), which were controlled by Analyst software (SCIEX). A Phenomenex reverse phase HPLC Omega (1.6 µm, PS C18, 100 × 2.1 mm, 100 Å) column with SecurityGuard™ ULTRA Cartridge was used for separation of the peptide mixtures resulting from trypsin digestion of CIC, CIM, and MIM whole cell extracts of hMDM, for analysis of intracellular galectins (2, 4, and 6 µg injections), or hMDM culture supernatants, for analysis of extracellular galectins (4 µg injection). A linear gradient program from 5 to 50% solvent B for 25 min was applied for the peptides mixtures separation at the flow rate of 0.2 mL/min. Then the gradient was increased to 95% B for 3 min and followed by column re-equilibration for 10 min, also at the flow rate of 0.2 mL/min. The solvent A was water containing 0.1% formic acid, while solvent B was ACN containing 0.1% formic acid (all LC–MS grade). The eluting peptides were ionized by ESI in TurboV Ion Source (SCIEX) at a positive 5 kV spray voltage and analyzed using MRM. The MRM transitions for galectin-1, -3, and -9 were in silico predicted using Skyline software (MacLean et al. 2010) and then empirically verified with the use of the pooled donors’ CIC-CIM-MIM hMDM sample. The parameters for the MRM transitions of the investigated galectins peptides are presented in the Table S1 (Supplementary Materials). Peak areas of the monitored MRM transitions from the LC-MRM raw data were extracted also using Skyline. They were used as an input data for the Excel package (Microsoft, Inc.) for calculation of their corresponding peptides, which were further calculated to the peak areas of the analyzed proteins. A two-sample unequal variance T-test was performed using the Log2 transformed protein peak areas to compare the investigated galectins levels between the studied conditions (CIC, CIM, and MIM). The differences were considered statistically significant when *p* < 0.05. The MRM dataset from our study is available in PASSEL repository (PeptideAtlas SRM Experiment Library) (Farrah et al. 2012) under the accession number PASS01591. Detailed description of the MRM assay development with Skyline settings for transition prediction and the method validation as well as the parameters of the LC-MRM method and statistical analysis are available in Macur et al. (2021).

### Immunofluorescence confocal microscopy analysis

Human monocytes were differentiated to macrophages as described above; on day 7 were exposed to Meth with the final concentration at 100 µM. Following 4-h incubation, cells were stained with LysoTracker® Red DND-99 (Thermo Fisher Scientific) according to the manufacturer’s protocol. Next, cells were fixed with cold 4% paraformaldehyde in phosphate-buffered saline PBS and permeabilized with 0.2% (w/v) Triton X-100 in phosphate-buffered saline (PBS) for 8 min. After washing with PBS, cells were stained with rabbit anti-galectin-1 (ab25138) (Abcam, Cambridge, United Kingdom) Abs (1:500), mouse anti-galectin-3 (B2C10) (Santa Cruz Biotechnology, Dallas, TX, USA) Abs (1:500) or rabbit anti-galectin-9 (ab69630) (Abcam) Abs (1:200) for 1.5 h. Next, cells were incubated with the suitable secondary antibodies Alexa 488-conjugated anti-mouse IgG (1:3000) or Alexa 488-conjugated anti-rabbit IgG (1:3000) (both Invitrogen) for 1 h. For cell nuclei visualization, ProLong™ Gold antifade reagent with DAPI (Invitrogen) was applied. Prepared slides were analyzed using a ZEISS LSM 800 confocal laser scanning microscope (Zeiss, Jena, Germany).

## Results

### Experimental design

Our main question in this study was the effect of Meth on already HIV-1-infected macrophages, and if such effect exists, how much macrophages are further altered in their intra- and extracellular metabolism. Figure 2 reflects such objective; thus, our control was set to be HIV-1-infected macrophages. In addition to full unbiased profiling, we focused on galectins, both intracellular and secreted. Another question asked was whether exposure to Meth prior to HIV-1 infection has a different effect on hMDM than exposed to Meth only after viral infection (CIM) or continuously (MIM).

### Analysis of gene expression

**Fig. 3:**
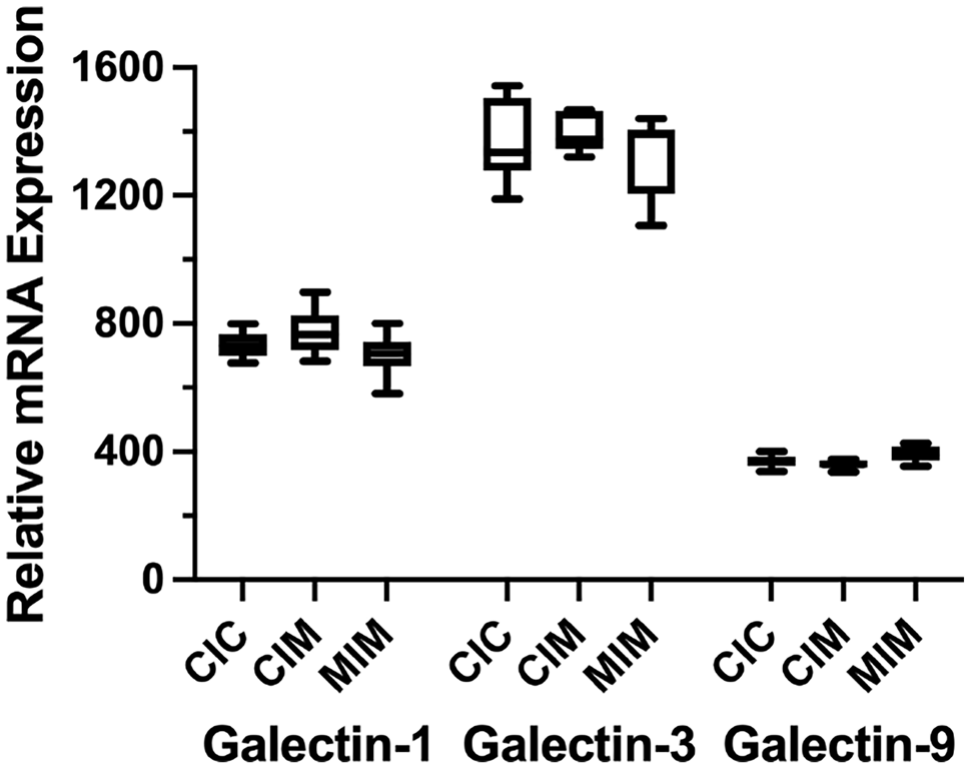
Box and whisker plots of RNA-seq determination of expression levels of galectin-1, -3, and -9. No change was found between the conditions

RNA was isolated from the cultures of six donors’ MDM, cultured under the three conditions, and subjected to RNA-seq. Examination of galectin-1, -3, and -9 expression levels did not reveal changes due to either Meth condition (Fig. 3).

### SWATH-MS analysis

As depicted in Fig. 2, our experimental design set HIV-1-infected hMDM (with no Meth treatment) as the control (CIC), experimental samples when Meth was added after viral infection as CIM, or both before and after infection as MIM. We applied nanoLC-SWATH-MS approach to obtain full unbiased proteomic profiles of hMDM in each of the studied conditions. This enabled us to see changes in the proteins’ expression on the global level that occur as a consequence of HIV infection with simultaneous Meth treatment pre- and/or-post infection. In the SWATH experiment, we used monocytes from five donors (five biological replicates) that were analyzed in five technical replicates (five injections of each sample). Details of this SWATH-MS experiment were reported previously (Macur et al. 2021). Briefly, a reference spectral library for SWATH data extraction was generated experimentally using DDA mass spectrometry. We used non-fractionated trypsin digest of pooled CIC, CIM, and MIM samples from four donors to generate a library that contained 1241 proteins (1% FDR). This library was then applied for SWATH data extraction. The obtained peak areas of proteins detected and quantified in our study for each of the investigated conditions (CIC, CIM, MIM) were then compared using *t* test and Log_10_(Fold Change) between CIC versus CIM, CIC versus MIM, and CIM versus MIM were calculated. Table 1 shows this relative quantification and consists of data extracted from SWATH-MS experiment specifically for galectins-1, -3, and -9.

**Table 1:** Differences in expression of intracellular galectins identified and quantified by SWATH experiment in hMDM in CIC, CIM, and MIM conditions

UniProt accession number	Name	CIC vs CIM		CIC vs MIM		CIM vs MIM	
		*p value*	*Log*_*10*_*(Fold Change)*	*p value*	*Log*_*10*_*(Fold Change)*	*p value*	*Log*_*10*_*(Fold Change)*
sp|P09382|LEG1_HUMAN	Galectin-1	6.40E-01	2.78E-02	4.39E-01	–5.87E-02	2.32E-01	–8.65E-02
sp|P17931|LEG3_HUMAN	Galectin-3	9.12E-01	–6.25E-03	8.00E-01	1.37E-02	4.43E-01	1.99E-02
sp|O00182|LEG9_HUMAN	Galectin-9	6.96E-01	–4.95E-02	3.68E-01	–1.77E-01	4.52E-01	–1.27E-01

### MRM analysis

To verify our findings from the SWATH experiment, we quantified galectins-1, -3, and -9 using LC-MRM, a well-established MS-based approach for reproducible and sensitive quantification. MRM-based quantification is a more precise and accurate method than full unbiased SWATH-MS. In some instances, SWATH-MS does not detect some proteins while MRM can provide reproducible quantification. In this study, we used an MRM approach to validate and a more in-depth mass spectrometry-based analysis to validate the expression of three galectins representing three sub-types. At this stage, we measured both intra- and extracellular galectins levels in hMDM whole cell protein extracts and cell supernatants, respectively. We analyzed CIC, CIM, and MIM samples separately for 5 donors (each in 5 technical replicates). We developed the MRM assays for the investigated galectins with the aid of in silico MRM transitions prediction using Skyline software (MacLean et al. 2010). The theoretical transitions were then verified experimentally on a pooled CIC-CIM-MIM sample, to ensure reliable analysis of the target samples. The LC-MRM methodology was also validated using serial dilutions of bovine serum albumin standard spiked into the CIC-CIM-MIM sample matrix. The MRM transitions monitored for the galectin-1, -3, and -9 are presented in Table S1 (Supplementary Material). The detailed information about LC-MRM assay development, validation, the LC–MS/MS settings, and data analysis is available in (Macur et al. 2021).

### Intracellular galectins

**Fig. 4:**
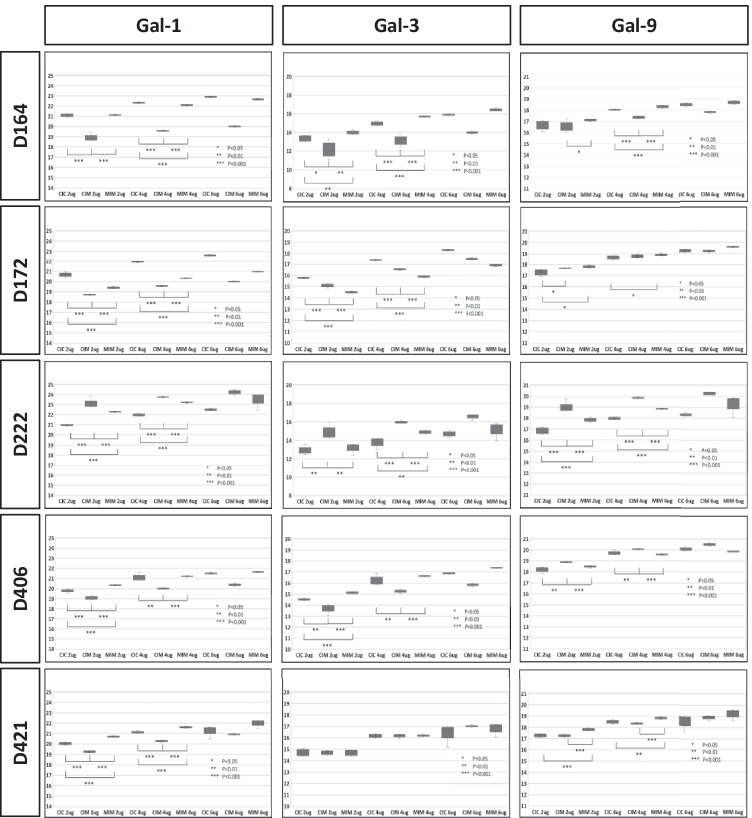
MRM quantification of intracellular expression of galectins − 1, − 3, and − 9 under Meth treatment of HIV-1 infected hMDM. For quantification of each galectin, we used 2, 4, or 6 ug of cell lysates representing pool of intracellular proteins. Each condition was measured five times (five technical replicates). One star represents p < 0.05, two stars represent p < 0.01, and three stars represent p < 0.001

Figure 4 shows MRM quantification-based effect of Meth on the intracellular galectins-1, -3, and -9. Our baseline is hMDM infected with HIV-1 without Meth (CIC). In this figure, we show results of quantification of galectins in hMDM from five donors. Four (D164, D172, D406, and D421) out of five donors show similar pattern of expression of Gal-1 in three experimental conditions. Only one donor (D222) shows a different pattern contributing to the variability of responses between donors, an effect observed by us in many experiments. Interestingly, Gal-3 does not show a consistent pattern among the same set of donors. Three (D164, D172, and D421) show similar patterns of Gal-9 expression, while two other donors (D406 and D222) show different patterns than the other three donors; however the pattern is similar for these two. Summarizing, the characteristics of protein expression in primary macrophages varies in different donors. We postulate those differences in patterns would be dependent on other factors, such as the donor and response to HIV infection. It has to be noted that samples for this study represent one time point, while the response of macrophages can be very dynamic in terms of quantity of changes. We also postulate that short-lived regulatory proteins might show highly diverse patterns.

### Extracellular galectins

**Fig. 5:**
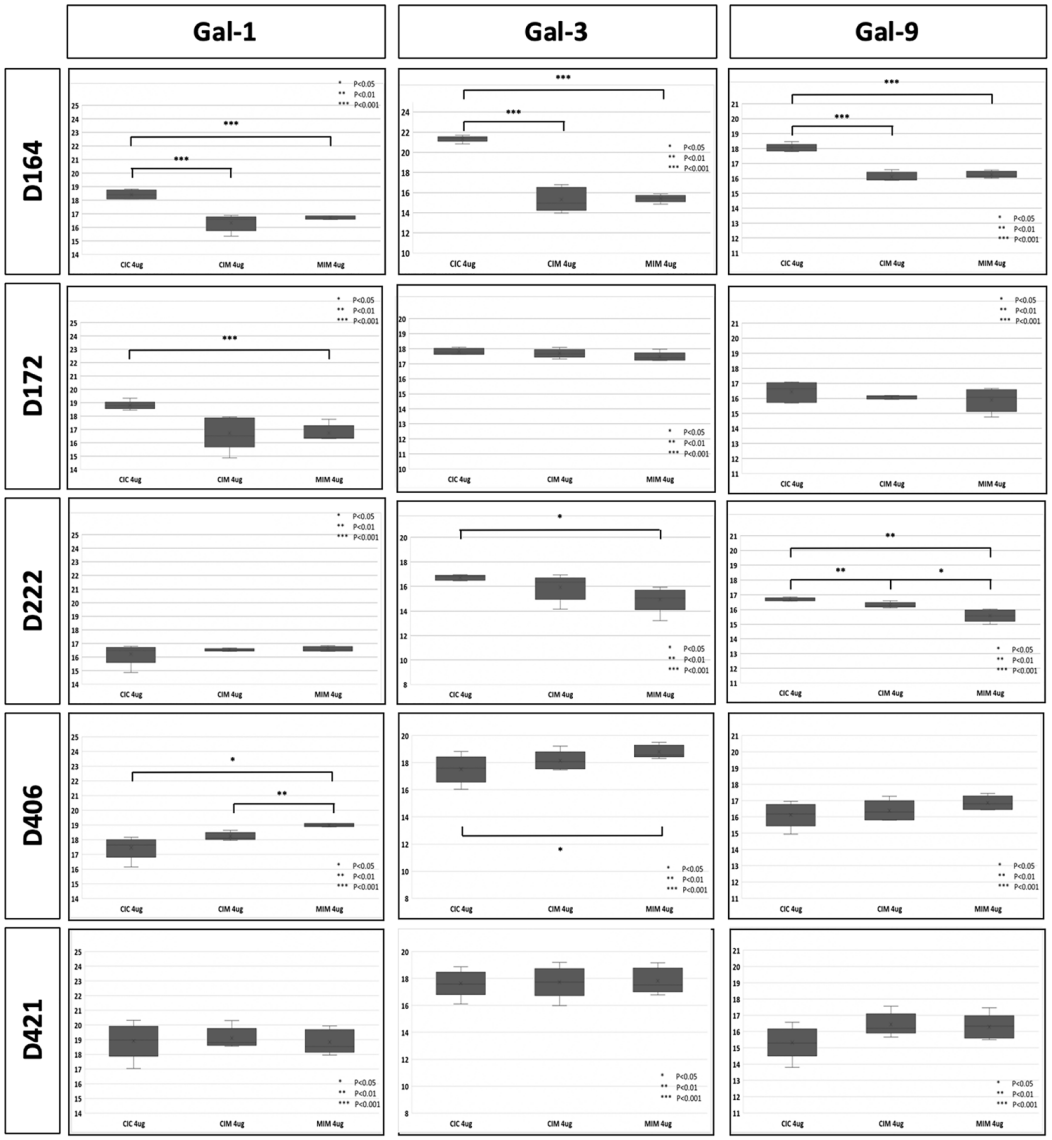
MRM quantification of extracellular expression of galectins-1, -3, and -9 under Meth treatment of HIV-1-infected hMDM. For quantification of each galectin, we used 4 ug of cell lysates representing a pool of extracellular proteins. Each condition was measured five times (five technical replicates) in cultures of hMDM from five donors. One star represents p < 0.05, two stars represent p < 0.01, and three stars represent p < 0.001

Concentrations of extracellular galectins are summarized in Fig. 5.

### Intra-cellular localization of galectins

**Fig. 6:**
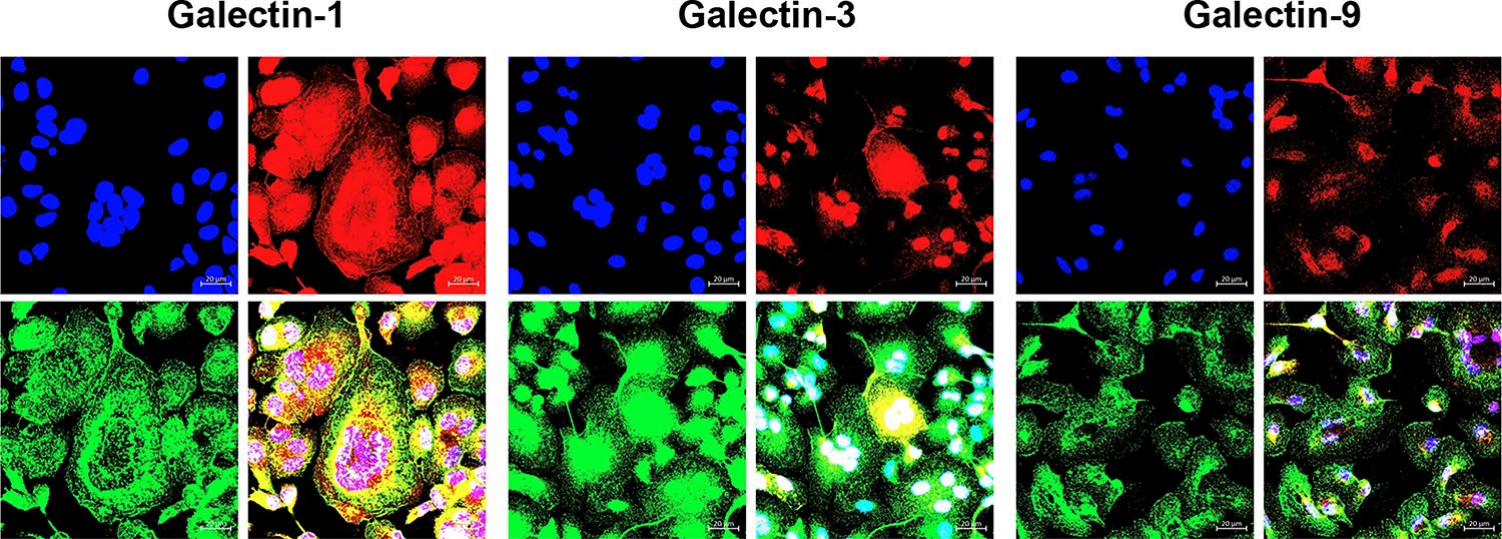
Intracellular localization of galectins-1, -3, and -9 in hMDM. Immuno-histostaining for CD163 (green), galectins (red), and nuclei DAPI (blue). Galectins are distributed across entire cells; nevertheless, most of them are around nuclei

As shown in Fig. 6, localization of galectins in cells under the control condition is across entire cytoplasm with a concentration in the perinuclear region. There is no significant difference between patterns of intracellular distribution of the three galectins being investigated here.

**Fig. 7:**
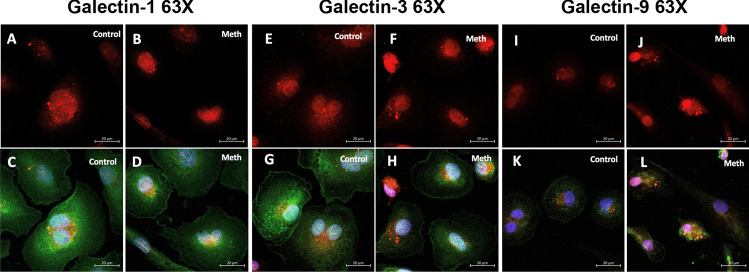
Cytostaining of control and Meth exposed hMDM for galectins -1, -3, and -9 (green), acidic organelles – lysosomes (fluorescently labeled by lysotracker—red), and nuclei (DAPI—blue). A, B, E, F, I, and J represent single channel for lysosomes; and C, D, G, H, K, and L are merged images

To test cellular distribution of galectins-1, -3, and -9 as well as colocalization with acidic organelles—lysosomes—cytostaining of control and Meth exposed hMDM was performed. In both conditions, galectins colocalize with acidic organelles and are observed in the central part of the cell. Interestingly, this effect is more pronounced for galectin-9 of hMDM Meth-treated (Fig. 7).

**Fig. 8:**
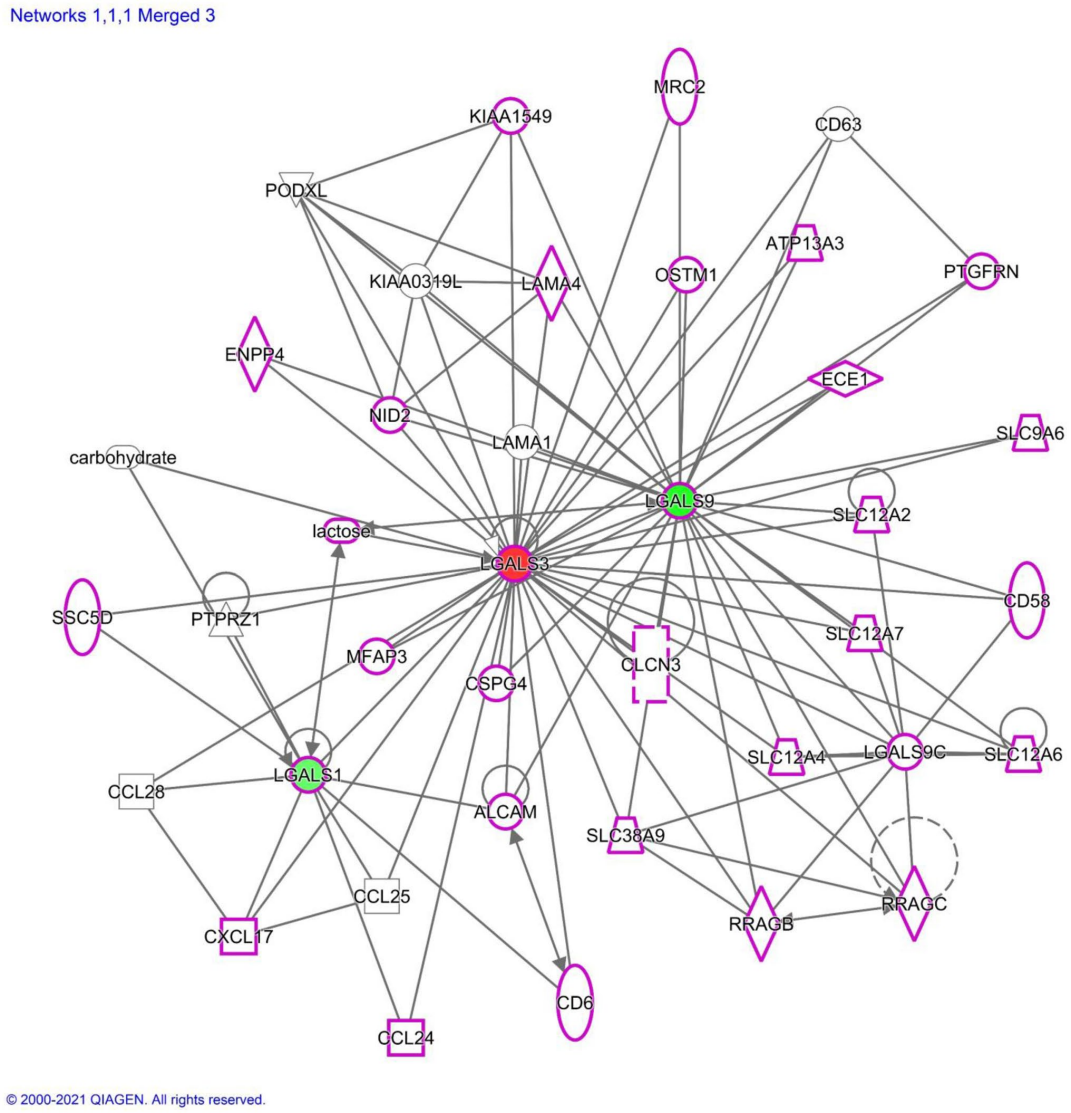
IPA network of galectins-1, -3, and -9 interactions. Red represents upregulated genes while green represents downregulated genes. The intensity of colors is directly proportional to the fold change. Purple shapes represent genes associated with lipid metabolism. The values are common number of genes between each observation

### Ingenuity pathway analysis (IPA)

**Fig. 9:**
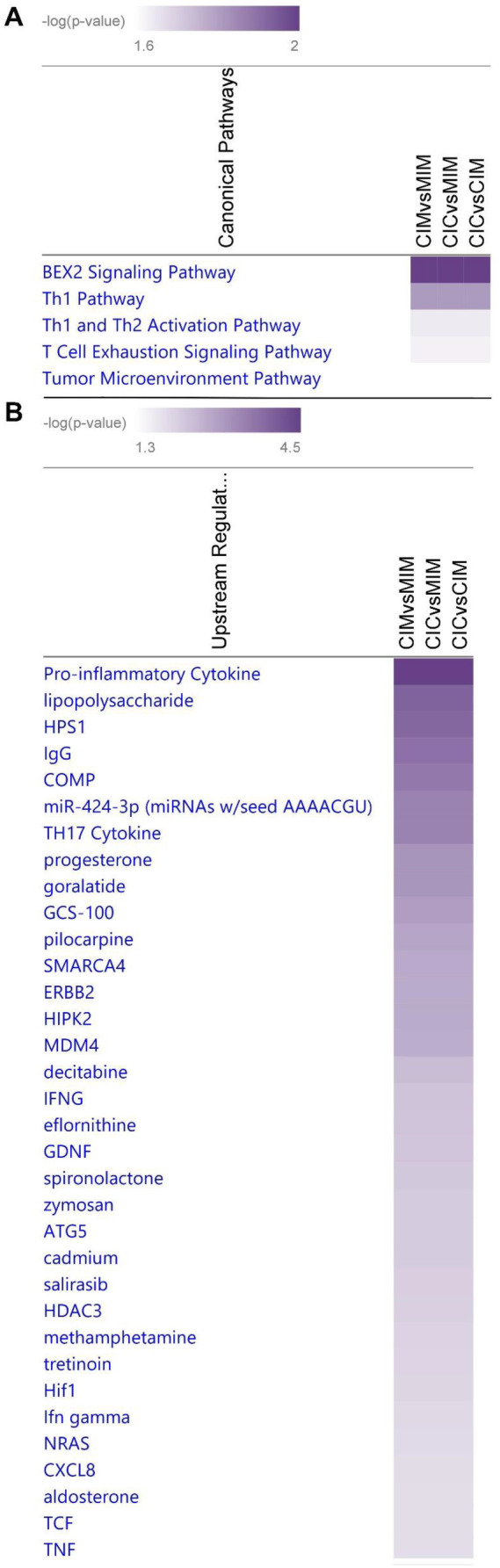
The results of the Ingenuity Pathways Core Analysis performed using Log_10_(Fold Change) values between CIC versus CIM, CIC versus MIM, and CIM versus MIM for galectins-1, -3, and -9 quantified in the nanoLC-SWATH-MS experiment. (**a**) Canonical pathways in which the investigated galectins are involved. (**b**) Upstream regulators that might be activated or inhibited to explain expression changes of the studied galectins. The color intensity ranges from the lowest to highest -log (p value)

Data obtained from MarkerView were used for comparisons of three biological conditions CIC vs CIM, CIC vs MIM, and CIM vs MIM and were uploaded to IPA as three separate observations. The data comprised of p-value and Log_10_(Fold Change) for each observation. Then, core analysis was run with preset analysis settings in which we specified analyzing all pathways for humans only. The results for all three observations were then run through comparison analysis in IPA. Insignificance threshold for –log(P-value) was set to 1.5 for the outputs obtained. The three networks were finally overlapped to find connections between galectin-1, -3, and -9 (Fig. 9).

IPA analysis presented in Fig. 8 shows connection of galectins-1, -3 and -9 in their regulatory processes. The presented network shows that these galectins are connected as well as they are connected to a number of other regulatory proteins. One group consists of several chemokines such as CXCL17, CCL24, CCL25, and CCL28 associated primarily with galectin-1. These chemokines are involved in regulating, in positive or negative mode, T cells, macrophages, monocytes, and dendritic cells (DC), all relevant for the function of the immune system (Lee et al. 2013) as well as other cells of hematopoietic origin.

The same network shows several genes involved with ion transport (Lysosomal Solute Carrier Transporters) such as SLC9A6, SLC12A2, SLC12A7, SLC12A6, SLC12A4, and SLC38A9 that are associated primarily with galectin-9. SLC transporters have been shown as essential for ion, molecule, and other solute transport to support lysosomal function (Bissa et al. 2016). More specifically SLC38A9 mediates transport of amino acids with low capacity and specificity with a slight preference for polar amino acids. SLC12A2 cation-chloride cotransporter mediates the electroneutral transport of chloride, potassium, and/or sodium ions across the membrane. SLC9A6 electroneutral exchange of protons for Na^+^ and K^+^ across the early and recycling endosome membranes contributes to calcium homeostasis. SLC12A7 mediates electroneutral potassium-chloride cotransport when activated by cell swelling. SLC12A6 mediates electroneutral potassium-chloride cotransport. It is important to note that our cyto-immune staining shows some association of galectins with LysoTracker; however, Meth does not have a profound effect on such co-localization.

Another example is ALCAM (activated leukocyte cell adhesion molecule, Q13740, CD166_HUMAN) that is directly connected to all galectins being investigated here (Ikeda and Quertermous 2004). ALCAM is a cell adhesion molecule mediating cell–cell contacts. Galectin-3 is located in the middle/center of IPA-generated network and link Gal-1 and Gal-9.

Further IPA core analysis showed that canonical analysis indicated the most change as measured by fold change was the BEX2 signaling pathway. BEX2 is a regulator of mitochondrial apoptosis and G1 cell cycle in breast cancer (Naderi et al. 2010). This aligns with other reports showing the effect of Meth on mitochondrial function (Barbosa et al. 2015). Following canonical pathways, we present upstream regulators. The latter category shows the strongest effect of Meth on pro-inflammatory cytokines (Fig. 9b). Again, it aligns with previously reported observation showing pro-inflammatory response of macrophages exposed to Meth (Bortell et al. 2015; Burns and Ciborowski 2016).

## Discussion

As much as galectins themselves were studied to some extent, the effect of Meth on their expression is represented by very few publications (Parikh et al. 2015; Reynolds et al. 2012b, 2007). Published results do not allow for more comprehensive interpretation due to a variety of different experimental models used. In this study, based on SWATH-MS, followed by MRM, we present results of the effect of Meth on intracellular expression of galectin-1, -3, and -9 in HIV-1-infected hMDM. Analytical measurements applied here appear to be precise enough to detect small but significant differences. Our previous study (Haverland et al. 2014) showed that Meth had profound and statistically significant effects on expression of nucleic acid binding and regulatory proteins in HIV-1 infected macrophages. In that study, we used 4 donors of hMDM and performed SWATH-MS analysis using four groups: control, HIV-1 infected, Meth exposed, and HIV-1 infected and exposed to Meth; thus, experimental design was different than in this current one. Re-analyses of previous data are now included as Fig. S1 and Table S1 in the supplemental material.

Comparing previously published results (Haverland et al. 2014) and current SWATH-MS and MRM-MS analysis results, we conclude that SWATH-MS is substantially less sensitive and precise as MRM. Major limitation of MRM is low throughput while high throughput is major strength of SWATH-MS.

Reports describing the effect of Meth on expression and potential function(s) of galectins appear to be imprecise due to a variety of experimental models and experimental designs used. Here, we provide for the first time a systematic quantification of intracellular as well extracellular galectins.

Galectins are produced and secreted by many cell types and several important regulatory functions have been associated with their presence in circulation (Brinchmann et al. 2018; Johannes et al. 2018). Galectins belong to a group of cytoplasmic proteins that can be secreted to the surrounding milieu even without signal sequence for secretion. Popa et al. propose four mechanisms of secretion of galectins (Popa et al. 2018) out of which three are based on vesicle transport. Since we observe that in the presence of Meth, whether for shorter (CIM) or longer (MIM) time of exposure, secretion of all galectins is inhibited. Since we do not observe significant suppression of mRNA (see Fig. 3), transport of galectins to the extracellular space can be linked to deregulation of Rab proteins that are de-regulated upon Meth exposure (Macur et al. 2021) or other proteins involved in the regulation of vesicle-mediated transport.

Galectins are postulated to be involved in multiple regulatory processes via recognition of glycoconjugates, some of which are related to the regulation of the immune system by affecting T cells, macrophages, and B cells. As reported by Sato et al. (Sato et al. 2012), presentation of complex glycans on HIV-1 gp120 glycoprotein can be recognized by galectins-1, -3, and -9. Proposed mechanism postulates that these galectins might be involved in increasing HIV-1 infectivity and transmission via recognition of oligomannosyl polysaccharides, though their specificity and exact molecular mechanism is not entirely clear.

Our studies show that Meth has quite a profound effect on the expression of these three galectins in hMDM and this effect depends on the time of exposure, CIM vs. MIM. We suggest that these differences reflect the mechanism(s) of how macrophages try to cope with toxic insults such as presence of Meth. The molecular mechanism is not clear at this time and may include some epigenetically driven mechanisms to be explored in future studies.

Galectin-1. It was postulated that galectin-1 increases HIV-1 infectivity of T cells and macrophages through a mechanism involving an initial interaction with gp120 (Ouellet et al. 2015). Galectin-1 is the only galectin studied in the presence of illicit drugs (Reynolds et al. 2012a, b). In works of Reynolds group, morphine was used as a model, and the vast majority of results were acquired from experiments investigating gene expression. Our data presented in Fig. 4 show intracellular expression of galectin-1 in hMDM at the protein level showing significant suppression of this protein due to Meth exposure in already HIV-1-infected hMDM. Although prolonged exposure to Meth shows a slight rebounded expression, it still remains significantly lower than in the absence of Meth.

Galectin-3. It has been postulated that increase of galectin-3 is induced by expression of Tat protein mediated by the Sp-1 binding transcription factor. This conclusion was made based on an investigation involving several transformed cell lines (Fogel et al. 1999) but not primary human cells, such as T cells and macrophages, which are direct targets of HIV-1. Our data presented in Fig. 4 show a profound decreased expression of intracellular galectin-3 particularly after longer exposure to Meth as it was in MIM condition. This appears opposed to observations presented by Fogel et al. postulating that expression of galectin-3 is related to the expression of gene *tat* (Fogel et al. 1999). Otherwise, we would expect that transcription and protein level of Tat would be increased during active infection of hMDM. Importantly, Lee and colleagues (Lee et al. 2002) did not observe an effect on SP-1 biding activity to DNA due to Meth injections into mice. Summarizing, it is not the first time when results from several studies show opposite results; therefore, galectins warrant further investigations because they are undoubtfully significant pathogenic factors in HIV-1 infections.

Galectin-9. The role of galectin-9 in viral infections, including HIV-1, appears multipurpose. Some reports suggest that galectin-9 plays a role as a negative regulator in T cells’ immune response to viral infections (Merani et al. 2015), while others postulate that galectin-9 is a potent mediator of HIV-1 transmission and reactivation (Abdel-Mohsen et al. 2016). It must be noted that all investigations reported to date are focused on T cells, while expression of galectin-9 as a pathogenic and/or regulatory factor was not investigated in macrophages. This makes our data presented in Fig. 5 a novel observation showing that when HIV-1-infected hMDMs are exposed to 100 µM of Meth, expression of galectin-9 is significantly increased despite whether Meth is added prior to or after viral infection. We may speculate that increased expression of galectin-9 in hMDM will translate, in part, to increased extracellular levels of this lectin in cerebrospinal fluid (CSF) and/or peripheral blood. This hypothesis has yet to be tested, and the significance of galectin-9 expression intracellularly in macrophages is not clear. Another question is whether galectin-9 may contribute to higher infectivity of macrophages by HIV-1 while exposed to Meth.

## Conclusions

Our data of comprehensive quantification of intra- and extracellular galectins uncover specific changes induced by Meth (Figs. 4 and 5). Interestingly, we did not observe significant effect of Meth at the RNAseq level. SWATH-MS analysis showed differerences in fold changes of galectin-1, -3 and -9, between CIC, CIM and MIM conditions, but they were not statistically significant.This is most likely due to a lack of sensitivity required to quantify small changes. As expected, MRM measurements showed more significant differences. Some differences are very small and exhibit significance close to borderline. Immune-cytostaining showed quite even distribution of galectins throughout cytoplasm with some overlap with lysosomes stained with LysoTracker. Further bioinformatic investigations show pro-inflammatory phenotype of hMDM and suggest a possibility of a much broader effect particularly in the local milieu of macrophages residing in tissues such as the brain (Figs. 8 and 9).

Thus, our study provides the first comprehensive quantification of three galectins representing three types of these proteins and should be considered as a foundation for future studies such as latency of HIV-1 infection. Further bioinformatic investigation of metabolic pathways combined with various experimental designs might be critical in deciphering the exact role of these important regulators of the innate immune system as potential biomarkers of disease progression and efficacy of therapies.

## Supplementary Information

Below is the link to the electronic supplementary material.

Supplementary file1 (DOCX 3132 KB)

## Data Availability

The mass spectrometry datasets used in this study were deposited to PRIDE (DDA and SWATH data – accession number PXD023291) or the PeptideAtlas PASSEL (MRM data – accession number PASS01591) repositories, which are both members of the ProteomeXChange Consortium. The RNAseq datasets have deposited in NCBI GEO and are available with accession number GSE160323.
