# Supplementary material for: HIV-1 and methamphetamine alter galectins -1, -3, and -9 in human monocyte-derived macrophages
Source: J Neurovirol. 2022 Feb 17;28(1):99–112. doi: 10.1007/s13365-021-01025-4 (PMC9076712; doi:10.1007/s13365-021-01025-4)

Journal of NeuroVirology

HIV-1 and Methamphetamine alters Galectins -1, -3, and -9 in Human Monocyte-Derived Macrophages

Supplementary Materials

Kinga Grabowska^1,2, $^, Katarzyna Macur^1,3, $^, Sarah Zieschang^1^, Lubaba Zaman^1^, Nicole Haverland^1^, Andrew Schissel^1^, Brenda Morsey^1^, Howard S. Fox^1^, and Pawel Ciborowski^1, *^

^1^ Department of Pharmacology and Experimental Neuroscience, School of Medicine, University of Nebraska Medical Center, Omaha, NE

^2^ Laboratory of Virus Molecular Biology, Intercollegiate Faculty of Biotechnology, University of Gdańsk and Medical University of Gdańsk, Poland

^3^ Core Facility Laboratories, Intercollegiate Faculty of Biotechnology, University of Gdańsk and Medical University of Gdańsk, Poland

*- Corresponding author

^$^ - Equal contribution

Dr. Pawel Ciborowski

Department of Pharmacology and Experimental Neuroscience

University of Nebraska Medical Center

985800 University of Nebraska Medical Center

Omaha, NE 68198-5800

Phone: 402-559-3733

Fax: 402-559-7495

Supplementary Materials


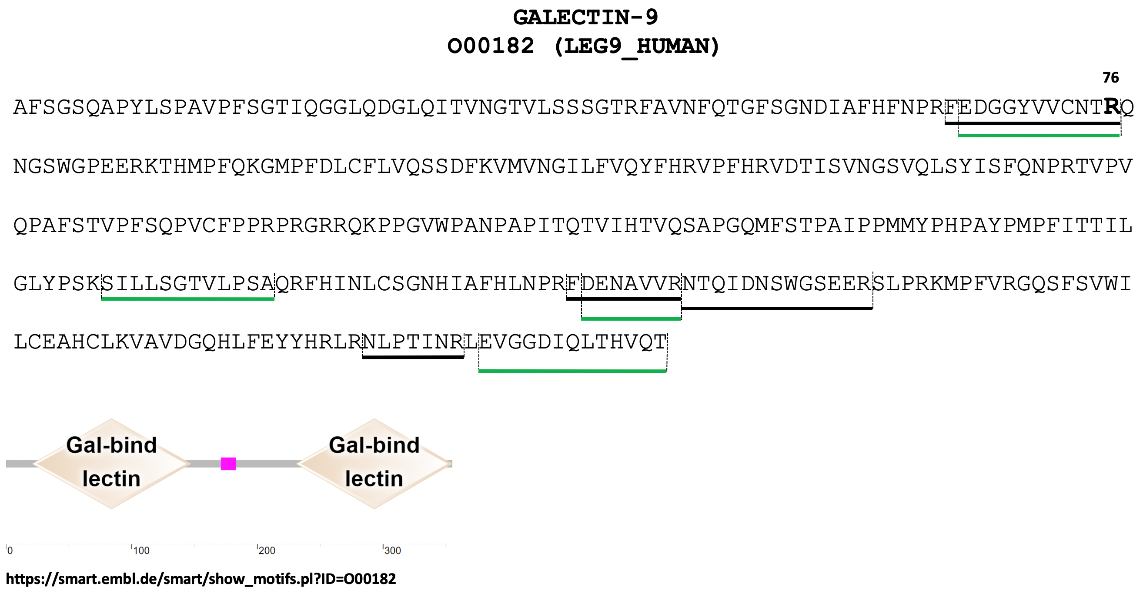

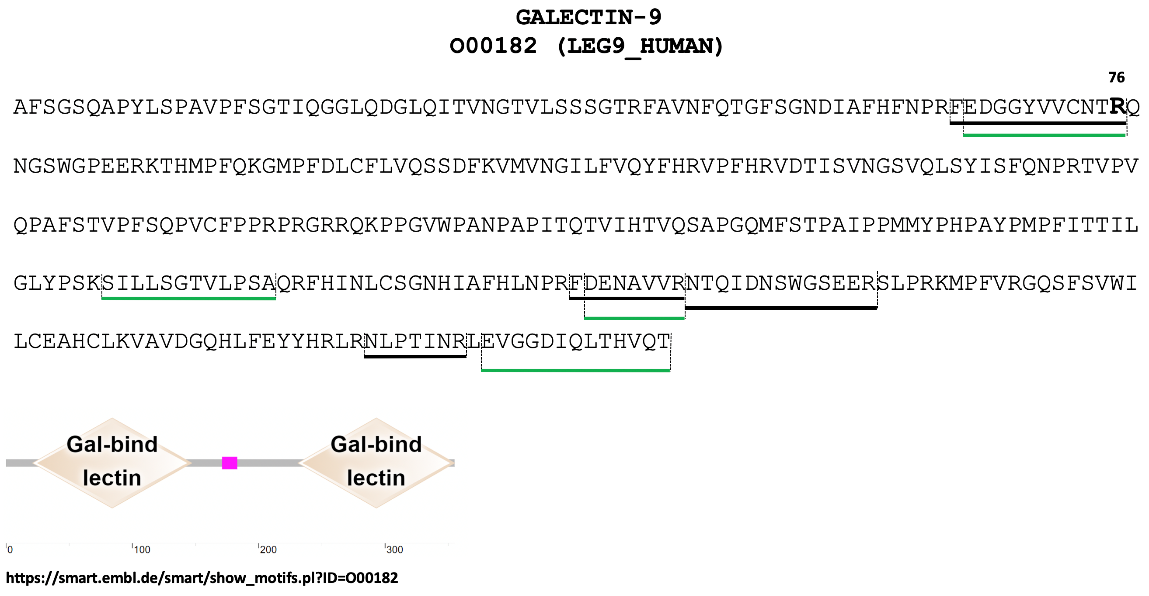

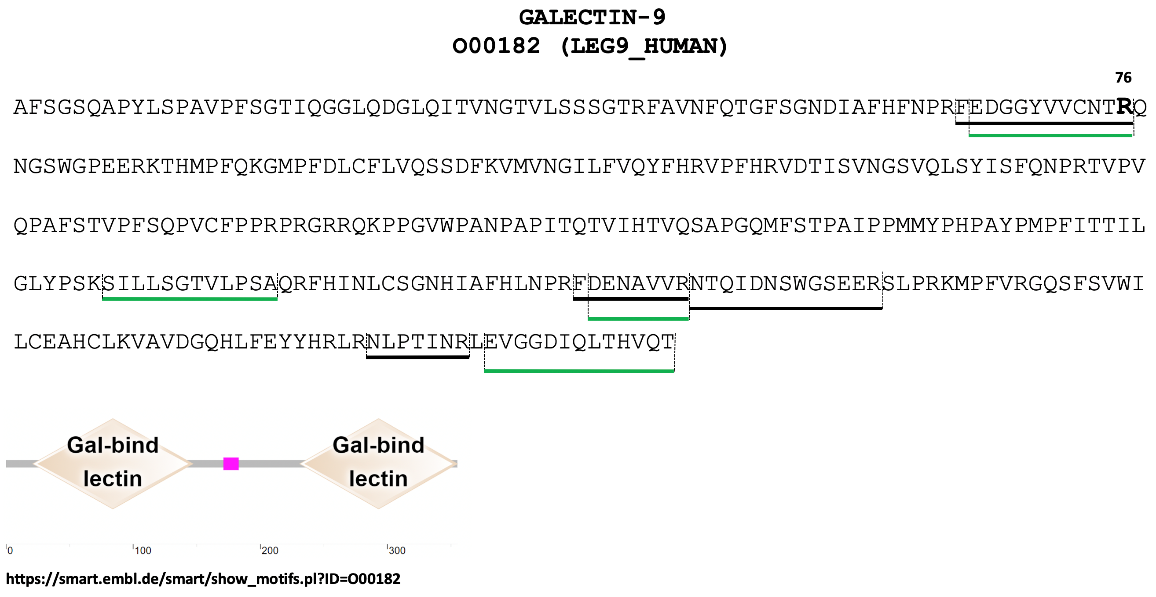

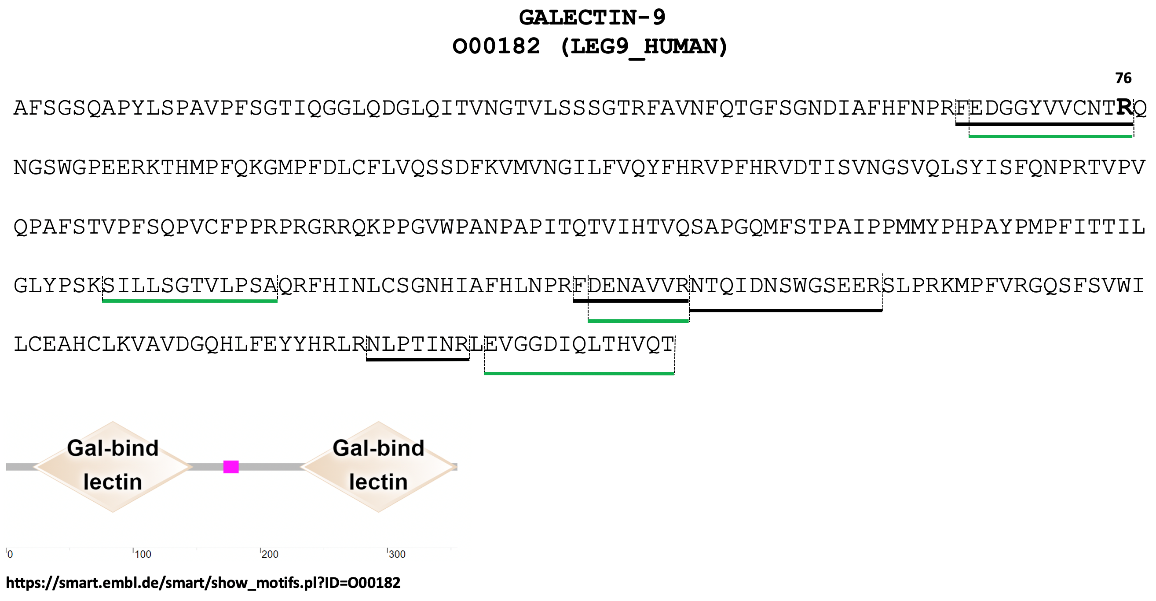

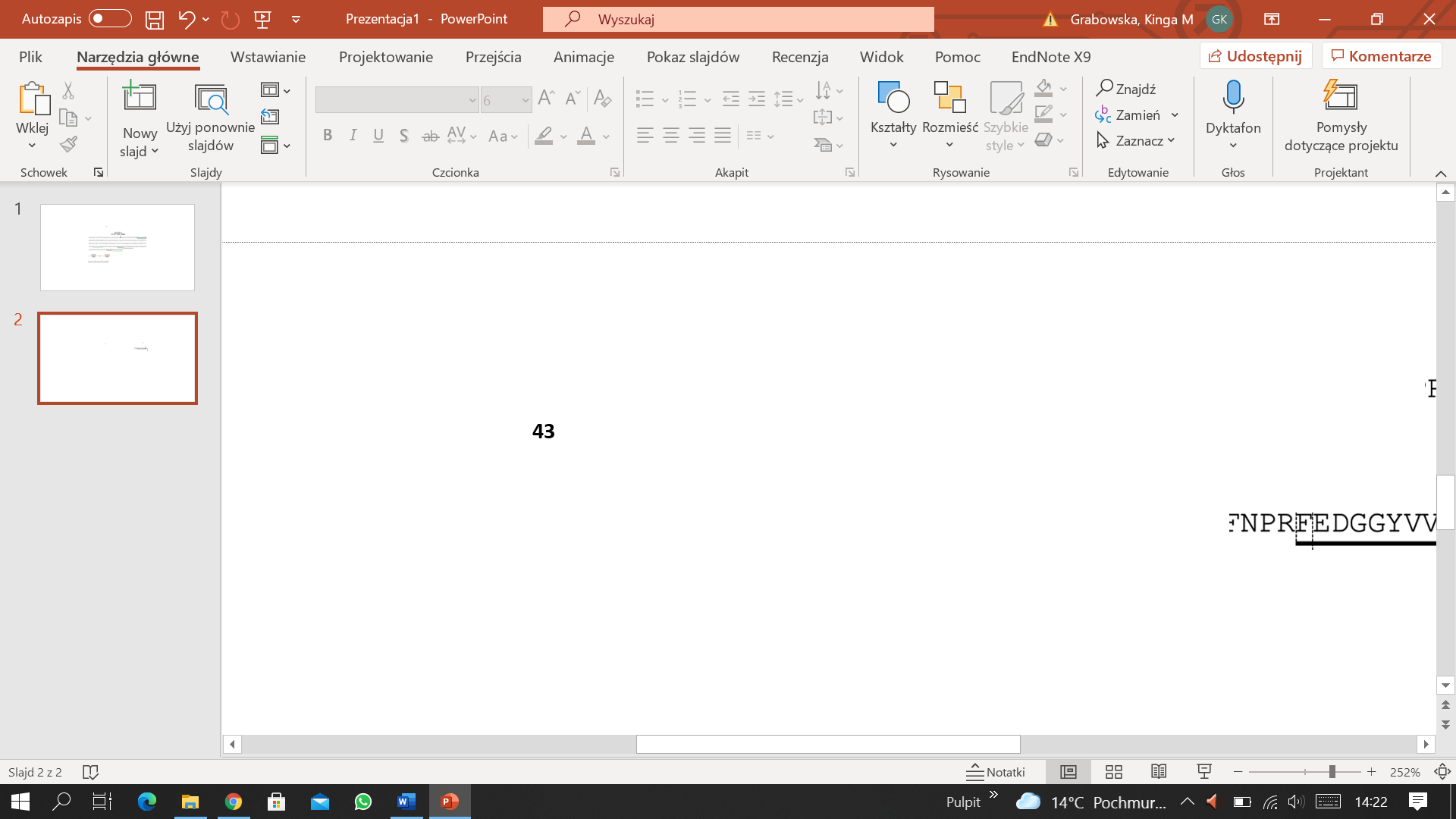


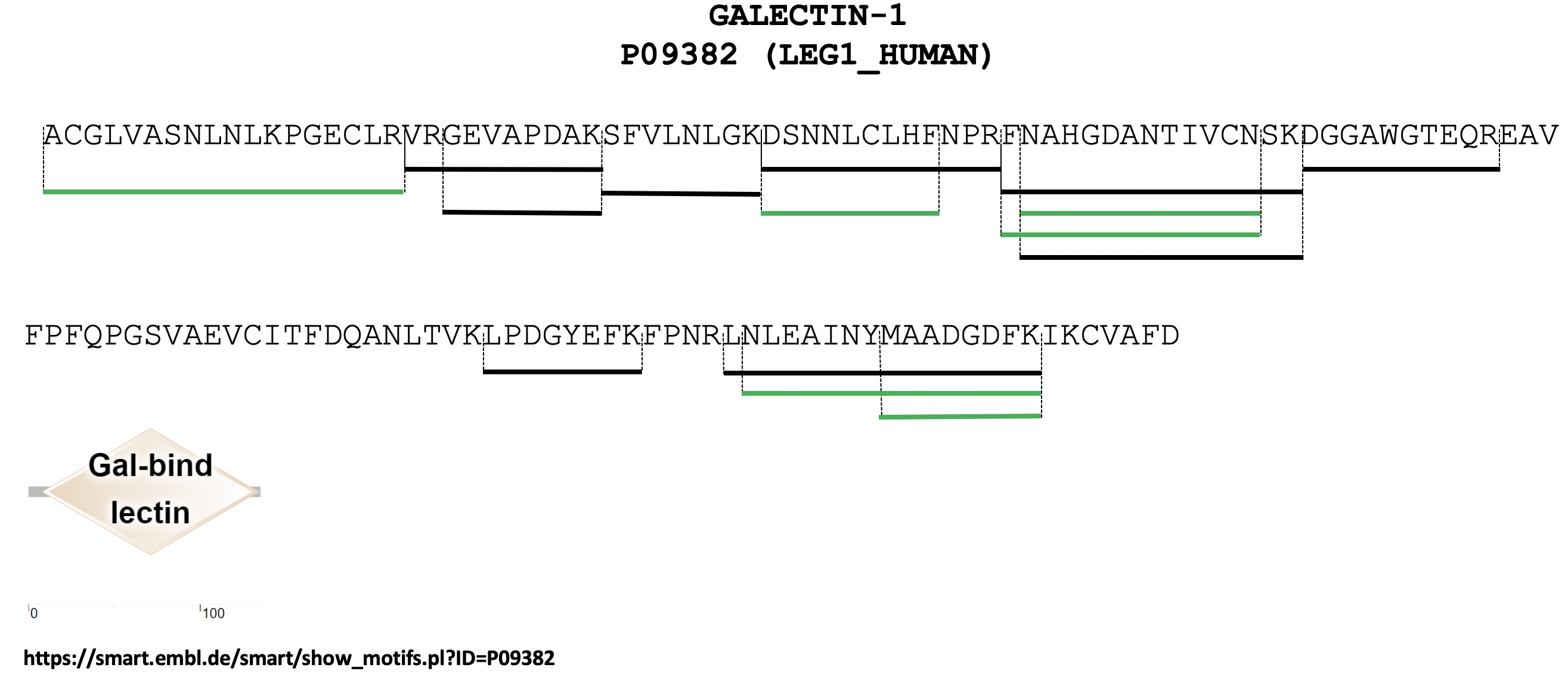


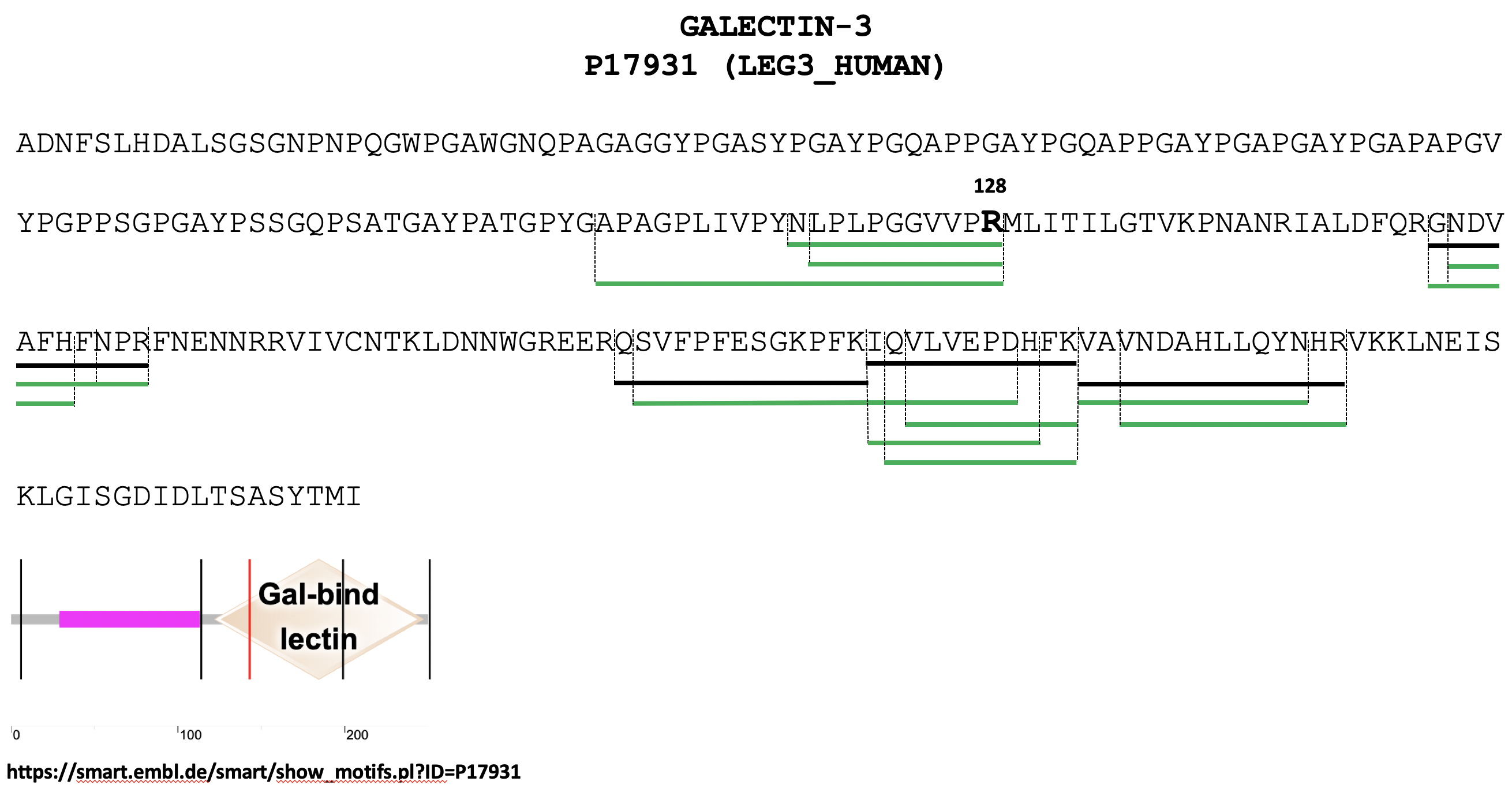


**Figure S1.** Sequence coverage of galectins -1, -3, and -9 obtained from MS analysis. Gal binding domain(s) were derived from <https://smart.embl.de/smart/show_motifs.pl?ID=O00182> and are shown under a.a. sequence. Black bars show peptides that are derived from sequence of K or R at position -1 of N-terminus and K or R at C-terminal position indicating that such peptides were derived from continuous sequence by trypsin. Green bars indicate peptides that have either K or R at position -1 of N-terminus and K or R at C-terminal position suggesting that these peptides were derived from fragment of galectins. For Gal -3, first R susceptible to trypsin cleavage is at position 128, while for Gal -9 it is at position 43. There is no sequence coverage of part located N-terminally to these arginine residues.


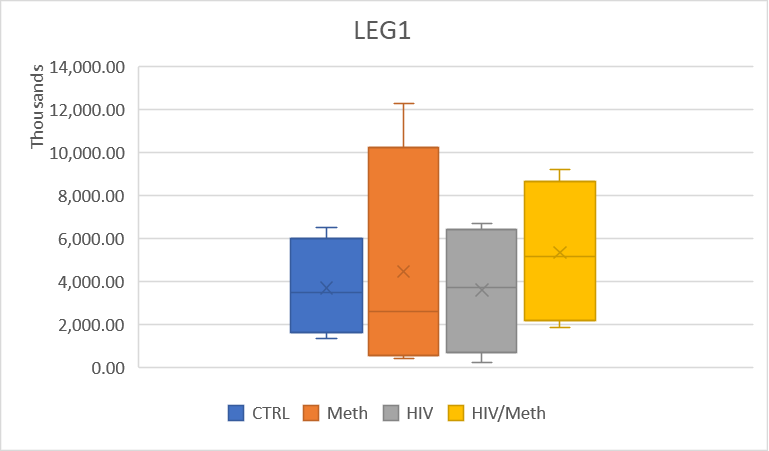

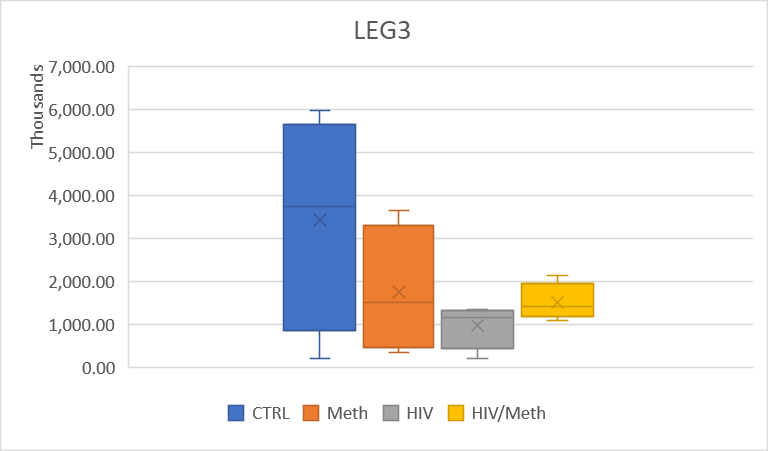

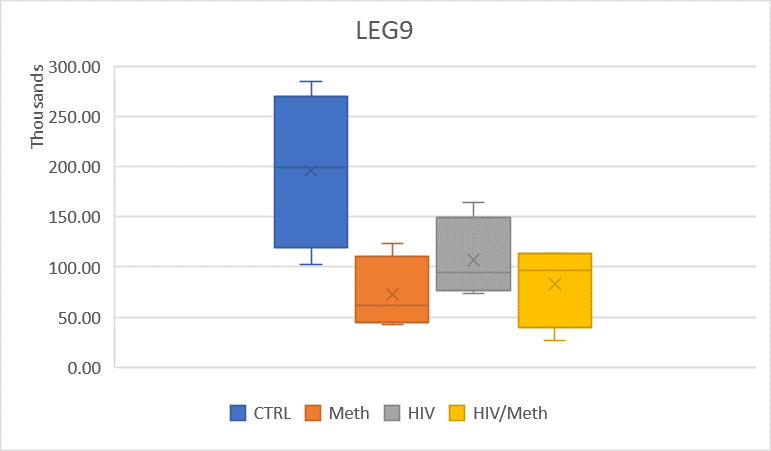


**Gal-1**

**Gal-3**

**Gal-9**


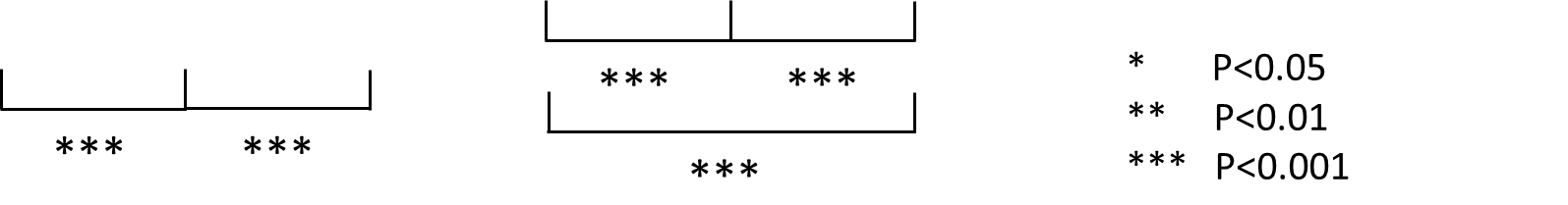


*

**Figure S2.** SWATH analysis of intracellular expression of Galectins -1, -3, and -9 in hMDM in untreated/uninfected (CTRL), Meth-treated (Meth), HIV-infected (HIV), and HIV-infected/Meth-treated (HIV/Meth) conditions. Each condition was measured for 4 donors. One star represents p-value < 0.05.

**Table S1.** The MRM transitions used for galectin-1, -3, and -9 quantitative analysis in CIC/CIM/MIM donors samples. Abbreviations used in the table: Q1 – precursor ion m/z, Q3 – fragment ion m/z, ID – transition name, DP – de-clustering potential, CE – collision energy, m/z – mass to charge ratio. Construction of the transitions IDs was as presented following the example of sp|P17931|LEG3_HUMAN.IQVLVEPDHFK.+2y7ID for peptide IQVLVEPDHFK from human galectin-3 (LEG3_HUMAN) protein: UniProt Accesion number (e.g. sp|P17931|) UniProt name (e.g. LEG3_HUMAN). Peptide amino acid sequence (e.g. IQVLVEPDHFK). Ion charge (e.g. +2) Fragment ion type (e.g. y7) Fragment ion charge (e.g. +1)

| **Q1** | **Q3** | **Dwell time** | **ID** | **DP** | **CE** |
| --- | --- | --- | --- | --- | --- |
| 393.7032 | 600.3352 | 25 | sp\|P09382\|LEG1_HUMAN.GEVAPDAK.+2y6 | 59.8 | 23 |
| 393.7032 | 501.2667 | 25 | sp\|P09382\|LEG1_HUMAN.GEVAPDAK.+2y5 | 59.8 | 23 |
| 393.7032 | 430.2296 | 25 | sp\|P09382\|LEG1_HUMAN.GEVAPDAK.+2y4 | 59.8 | 23 |
| 484.7398 | 855.3883 | 25 | sp\|P09382\|LEG1_HUMAN.LPDGYEFK.+2y7 | 66.5 | 26.3 |
| 484.7398 | 758.3355 | 25 | sp\|P09382\|LEG1_HUMAN.LPDGYEFK.+2y6 | 66.5 | 26.3 |
| 484.7398 | 428.1978 | 25 | sp\|P09382\|LEG1_HUMAN.LPDGYEFK.+2y7+2 | 66.5 | 26.3 |
| 892.9298 | 1131.478 | 25 | sp\|P09382\|LEG1_HUMAN.LNLEAINYMAADGDFK.+2y10 | 96.2 | 41 |
| 892.9298 | 854.3713 | 25 | sp\|P09382\|LEG1_HUMAN.LNLEAINYMAADGDFK.+2y8 | 96.2 | 41 |
| 892.9298 | 723.3308 | 25 | sp\|P09382\|LEG1_HUMAN.LNLEAINYMAADGDFK.+2y7 | 96.2 | 41 |
| 637.3073 | 888.4475 | 25 | sp\|P17931\|LEG3_HUMAN.GNDVAFHFNPR.+2y7 | 77.6 | 31.8 |
| 637.3073 | 817.4104 | 25 | sp\|P17931\|LEG3_HUMAN.GNDVAFHFNPR.+2y6 | 77.6 | 31.8 |
| 637.3073 | 670.342 | 25 | sp\|P17931\|LEG3_HUMAN.GNDVAFHFNPR.+2y5 | 77.6 | 31.8 |
| 662.8666 | 1083.583 | 25 | sp\|P17931\|LEG3_HUMAN.IQVLVEPDHFK.+2y9 | 79.4 | 32.7 |
| 662.8666 | 984.5149 | 25 | sp\|P17931\|LEG3_HUMAN.IQVLVEPDHFK.+2y8 | 79.4 | 32.7 |
| 662.8666 | 871.4308 | 25 | sp\|P17931\|LEG3_HUMAN.IQVLVEPDHFK.+2y7 | 79.4 | 32.7 |
| 878.9373 | 1101.513 | 25 | sp\|P17931\|LEG3_HUMAN.LGISGDIDLTSASYTMI.+2y10 | 95.2 | 40.5 |
| 878.9373 | 986.4863 | 25 | sp\|P17931\|LEG3_HUMAN.LGISGDIDLTSASYTMI.+2y9 | 95.2 | 40.5 |
| 878.9373 | 772.3546 | 25 | sp\|P17931\|LEG3_HUMAN.LGISGDIDLTSASYTMI.+2y7 | 95.2 | 40.5 |
| 475.2405 | 687.3784 | 25 | sp\|O00182\|LEG9_HUMAN.FDENAVVR.+2y6 | 65.8 | 26 |
| 475.2405 | 558.3358 | 25 | sp\|O00182\|LEG9_HUMAN.FDENAVVR.+2y5 | 65.8 | 26 |
| 475.2405 | 373.2558 | 25 | sp\|O00182\|LEG9_HUMAN.FDENAVVR.+2y3 | 65.8 | 26 |
| 867.4234 | 914.4155 | 25 | sp\|O00182\|LEG9_HUMAN.VAVDGQHLFEYYHR.+2y6 | 94.4 | 40.1 |
| 867.4234 | 638.3045 | 25 | sp\|O00182\|LEG9_HUMAN.VAVDGQHLFEYYHR.+2y4 | 94.4 | 40.1 |
| 867.4234 | 675.3229 | 25 | sp\|O00182\|LEG9_HUMAN.VAVDGQHLFEYYHR.+2y10+2 | 94.4 | 40.1 |
| 755.399 | 1168.596 | 25 | sp\|O00182\|LEG9_HUMAN.LEVGGDIQLTHVQT.+2y11 | 86.2 | 36.1 |
| 755.399 | 826.4417 | 25 | sp\|O00182\|LEG9_HUMAN.LEVGGDIQLTHVQT.+2y7 | 86.2 | 36.1 |
| 755.399 | 634.3357 | 25 | sp\|O00182\|LEG9_HUMAN.LEVGGDIQLTHVQT.+2y12+2 | 86.2 | 36.1 |

**Table S2.** Differences in expression of intracellular galectins identified and quantified by SWATH experiment in hMDM in untreated/uninfected (CTRL), Meth-treated (Meth), HIV-infected (HIV) and HIV-infected/Meth-treated (HIV/Meth) conditions. Statistically significant values are in red.


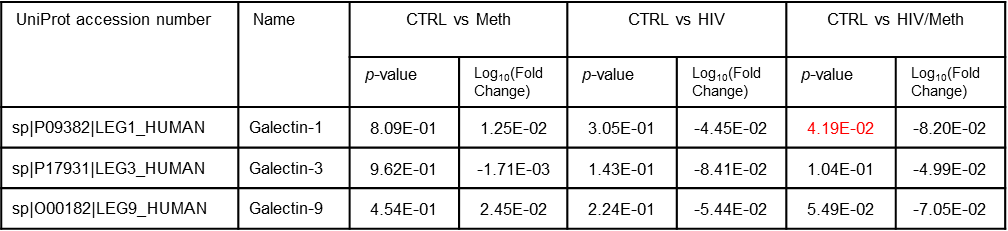

Supplement: Supplementary file 1 — Supplementary file1 (DOCX 3132 KB) [file 13365_2021_1025_MOESM1_ESM.docx]
